# The Role of Sphingolipid Metabolism and Neuron Death in Ischemic Stroke: A New Perspective from Bioinformatics

**DOI:** 10.1002/brb3.71172

**Published:** 2025-12-31

**Authors:** Zhong‐can Chen, Fang‐biao Xu, Sen Hu, Qiang Cui, Hugo Andrade Barazarte, Jing Zhang, Li‐na Suo, Jian‐jun Gu, Jiang‐yu Xue

**Affiliations:** ^1^ Department of Neurosurgery, Juha International Center for Neurosurgery, Henan Provincial People's Hospital, People's Hospital of Zhengzhou University People's Hospital of Henan University Zhengzhou Henan China; ^2^ Stroke Center, Cerebrovascular Disease Hospital Henan Provincial People's Hospital Zhengzhou Henan China; ^3^ Encephalopathy Center of The First Affiliated Hospital of Henan University of Chinese Medicine Zhengzhou China; ^4^ Collaborative Innovation Center of Prevention and Treatment of Major Diseases by Chinese and Western Medicine Zhengzhou China; ^5^ Institute for Brain Disorders of Henan Academy of Chinese Medical Sciences Zhengzhou China; ^6^ Department of Medical Records, Henan Provincial People's Hospital People's Hospital of Zhengzhou University, People's Hospital of Henan University Zhengzhou Henan China; ^7^ Department of Surgery, Division of Neurosurgery University Health Network (UHN) Toronto Canada

**Keywords:** ischemic Stroke, monocyte, neuron death, scRNA, sphingolipid metabolism

## Abstract

**Background:**

Ischemic stroke (IS) is a leading cause of death and disability worldwide, but traditional risk factors do not fully explain its pathophysiology. Neuronal death in IS is influenced by multiple pathways, including sphingolipid metabolism, which plays a significant role in neuronal function and survival. Ceramides, key sphingolipid molecules, are involved in various neuronal processes, including cell death. This study aims to explore the relationship between sphingolipid metabolism and neuron death in IS using bulk and single‐cell transcriptomics.

**Methods:**

We obtained sphingolipid metabolism gene sets from the GeneCard database and analyzed differential gene expression in IS datasets from the GEO database, including human peripheral blood bulk data (GSE16561) and MCAO mouse peripheral blood scRNA sequencing data (GSE225948). Gene set enrichment analysis (GSEA), immune infiltration analysis using CIBERSORT, and protein‐protein interaction network construction were performed. Single‐cell RNA sequencing (scRNA‐seq) data were used to identify key genes and analyze cellular heterogeneity, differentiation, and cell interactions. In vivo validation of key gene expression was conducted in MCAO rats.

**Results:**

GSEA revealed significant changes in the sphingolipid metabolism pathway in IS patients. Immune infiltration analysis showed altered immune cell profiles, with decreases in CD8 T cells and increases in monocytes and neutrophils. Enrichment analysis of sphingolipid metabolism‐related genes highlighted pathways such as the sphingolipid signaling pathway and ceramide metabolism. Protein‐protein interaction network analysis identified 19 key genes linked to sphingolipid metabolism and neuron death. scRNA‐seq analysis revealed significant changes in sphingolipid metabolism in monocytes and neutrophils, with the App gene showing notable differential expression. Pseudotime analysis suggested diverse differentiation trajectories in monocytes, and cell interaction analysis indicated potential communication between monocytes and B cells. In vivo validation confirmed higher App gene expression in MCAO rats compared to sham controls.

**Conclusion:**

This study provides comprehensive insights into the role of sphingolipid metabolism in ischemic stroke, identifying key genes and cellular mechanisms involved in neuron death. The findings suggest that sphingolipid metabolism, particularly through the App gene, may be a potential therapeutic target for IS. Further exploration of the molecular mechanisms and cellular interactions involving sphingolipids could lead to novel therapeutic strategies for ischemic stroke.

## Graphical abstract

First, we evaluated of differences in sphingolipid metabolism using peripheral blood samples from IS patients and healthy controls. Then, we identify the hub genes linking sphingolipid metabolism and neuronal death via modular analysis of protein‐protein interaction networks. Next, we verify the expression differences in hub genes linking sphingolipid metabolism and neuronal death using scRNA and evaluate the sphingolipid metabolism in cells. Finally, we explore the App gene function and validate the expression of the App gene in vivo.

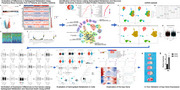

## 1 Introduction

Ischemic stroke (IS), according to the 2022 report by the American Heart Association, affects approximately 79,500 people annually in terms of stroke incidence in the U.S., with about 61,000 being first‐time cases and 185,000 being recurrent cases (Tsao et al. 2022). It ranks as the second leading cause of death and the third leading cause of disability among adults globally (Campbell and Khatri, 2020). However, traditional risk factors such as hypertension and diabetes alone cannot fully explain the epidemiological and pathophysiological characteristics of all cases, highlighting the necessity to identify additional potential risk factors and therapeutic targets.

The extent of damage from ischemic stroke largely depends on the number of neurons affected by ischemia‐related death in the impacted brain areas. Ischemia and reperfusion of brain tissue can trigger multiple pathogenic pathways, including bioenergetic failure, loss of cellular ion homeostasis, excitotoxicity, impairment of mitochondrial function, and the production of reactive oxygen species. These pathways are considered core mechanisms of neuron death in ischemic stroke. However, the significant role of lipids in neuron death during ischemic stroke has often been overlooked.

In mammals, the brain is the organ with the highest lipid content outside of adipose tissues. Sphingolipids are characteristic compounds of neuronal membranes. Crucial neurological functions, including information pathways and conduction, occur along these membranes. Therefore, it is unsurprising that neuronal function and survival depend on the metabolism of these lipids (van Echten‐Deckert and Alam 2018). Ceramides, as key molecules in sphingolipid and lipid metabolism and also as secondary messengers, affect various aspects of neuronal physiology, including cell proliferation, growth, survival, aging, and death (Maceyka and Spiegel 2014).

Single‐cell transcriptomics allows us to gain deep insights into the gene expression patterns of individual cells. It enables the revelation of cellular heterogeneity and diversity, exploring unique characteristics and functions of cells. This technology significantly aids in studying the molecular mechanisms behind disease development. It can also help in understanding abnormal expression patterns of cells under disease conditions, providing new clues for disease diagnosis and treatment. Single‐cell transcriptomics brings tremendous benefits to the life sciences, allowing us to study gene expression at higher resolutions and precision, thus advancing our understanding of biology and medicine (Zhang et al. 2023).

Ceramide is a representative compound in current sphingolipid research, sufficiently demonstrating the close link between sphingolipid metabolism and neuron death, which plays a crucial role in IS. Therefore, by integrating advanced scientific technologies, we conduct a comprehensive analysis based on bulk transcriptomics and single‐cell transcriptomics to thoroughly explore the connection between sphingolipid metabolism and neuron death in IS.

## 2 Methods

### 2.1 Data Source and Differentially Expressed Genes

We searched the GeneCard database (https://www.genecards.org/) using “Sphingolipid Metabolism, SPM” to obtain sphingolipid metabolism gene sets with a relevance score above 1. The GEO database was searched using “ischemic stroke” to obtain the human peripheral blood bulk chip expression profile GSE16561, which includes 39 ischemic stroke patients and 24 healthy controls, as well as the MCAO mouse peripheral blood single‐cell sequencing dataset GSE225948. Differential analysis of the chip data was performed using the limma‐trend method (Zhang et al. 2023). Probe sets that did not have a corresponding gene symbol were eliminated, and genes with multiple probe sets were averaged. Differential genes were those with a *p*‐value < 0.05 and |fold change| ≥ 1.

### 2.2 Gene Set Enrichment Analysis

In this study, the R package GSEABase (Chi et al. 2023) was used to perform gene set enrichment analysis (GSEA) on the sphingolipid metabolism gene set in the bulk dataset.

### 2.3 Immune Infiltration Analysis

To further analyze the immune microenvironment in the peripheral blood of IS patients, we used the GSE16561 dataset. The CIBERSORT algorithm (Newman et al. 2015), based on the principles of linear support vector regression, was employed to deconvolute the expression matrix of human immune cell subtypes and analyze immune cell infiltration. The limma package was used to analyze the differences in immune cells between the IS and control groups.

### 2.4 Identification of Hub Genes in SPM

This study utilized the R package VennDiagram (Chen and Boutros 2011) to analyze and plot a Venn diagram between the differential genes of the bulk dataset and the SPM gene set, defining the intersecting genes as SPM‐related genes.

### 2.5 Protein–Protein Interaction Network Construction and Module Analysis

The study obtained the protein interaction network between SPM‐related genes using the STRING (Szklarczyk et al. 2023) database (https://cn.string‐db.org/). This database extensively provides protein interactions for the submitted gene set. Subsequently, module analysis of the interaction network was performed using the walktrap community algorithm in the R package igraph (Saini et al. 2017), followed by GO biological process enrichment analysis for each module to further analyze the key biological processes of hub genes.

### 2.6 Enrichment Analyses of SPM‐Related Genes

The R package clusterProfiler (Zhang et al. 2023) was applied to perform gene ontology (GO) and Kyoto Encyclopedia of Genes and Genomes (KEGG) enrichment analysis on SPM‐related genes. GO and KEGG are among the largest gene annotation databases available. KEGG, for instance, helps analyze potential signaling pathways activated by a given gene set and can effectively assist in analyzing the potential molecular mechanisms of genes.

### 2.7 Quality Control of scRNA Datasets

The Seurat package was used for the quality control of single‐cell data. Samples with fewer than 200 nFeatures, mitochondrial gene expression over 10%, and ribosomal gene expression over 20% were excluded. This approach effectively eliminates potential factors such as doublets that may affect the analysis results. After PCA dimensionality reduction, batch effect removal with harmony, selection of appropriate PCA based on PCA results, and further dimensionality reduction with UMAP, a single‐cell data matrix was obtained. Manual annotation of scRNA data was performed using cell markers applied by the original data authors in their publication (Garcia‐Bonilla et al. 2024).

### 2.8 Expression Differences in the SPM Gene Set

The AddModuleScore function (Tirosh et al. 2016) in the Seurat package was used to score the SPM gene set, and the stat_compare_means function with the Wilcoxon test was used to analyze expression differences between cells and between MCAO and SHAM.

### 2.9 Expression Differences of Hub Genes

The stat_compare_means function with the Wilcoxon test in the ggpubr (Cheng et al. 2021) package was used to analyze the expression differences of hub genes between cells and between MCAO and SHAM. Based on changes in each cell type in MCAO and SHAM tissues and the expression level changes of hub genes, a specific cell type was selected for further analysis.

### 2.10 Impact of Hub Genes in Key Cells

To more accurately explore differences in signaling pathways activated by the varying expression levels of hub genes in key cells, the study divided the cells into high and low expression groups based on the median expression of hub genes. Utilizing the HALLMARK dataset, the R package irGSEA (Zhang et al. 2023) was used to calculate the differences in pathway activation between the two groups.

### 2.11 Pseudotime Analysis

To further understand the role of hub genes in the differentiation process of key cells, pseudotime analysis was performed on key cells using the monocle2 package (Qiu et al. 2017). Key gene group information was annotated, and BEAM analysis was performed on key branching points, with visualization of the biological processes of each fate.

### 2.12 Cell Chat

To further understand interactions between key cells and other cells, the CellChat package (Jin et al. 2021) was used for analyzing interactions between key cells and other cell types.

### 2.13 In Vivo Experimental Verification

SPF grade Sprague Dawley rats (weighing 280–300 g) were used in the experiment, with animal certificate number 370726231100699867, which was approved by the Ethics Committee of Henan University of Traditional Chinese Medicine (Approval No. IACUC‐202305042). The MCAO/R model (25) was used to simulate conditions in experimental rats, omitting the cerebral artery wire occlusion block in the Sham group. Post 24‐h modeling, a Longa Score (25) was performed with scores ranging from 1 to 3 chosen as the model group. Three animals each in the MCAO group and Sham group, at the end of a 7‐day period of raising, had blood from the abdominal aorta extracted and TTC staining conducted on the brain.

RNA was extracted from rat serum using the Trizol kit, with reverse transcription performed using the Takara kit. Real‐time fluorescent polymerase chain reaction (RT‐qPCR) involved setting up a reverse transcription system, executing the RT‐qPCR reaction, and calculating target gene mRNA expression levels. (Ren et al. 2023). Primers for detection of App can be found in Supplement Table 1. Difference analysis uses Wilcox tests.

## 3 Results

### 3.1 Data Source and Differentially Expressed Genes

**FIGURE 1:**
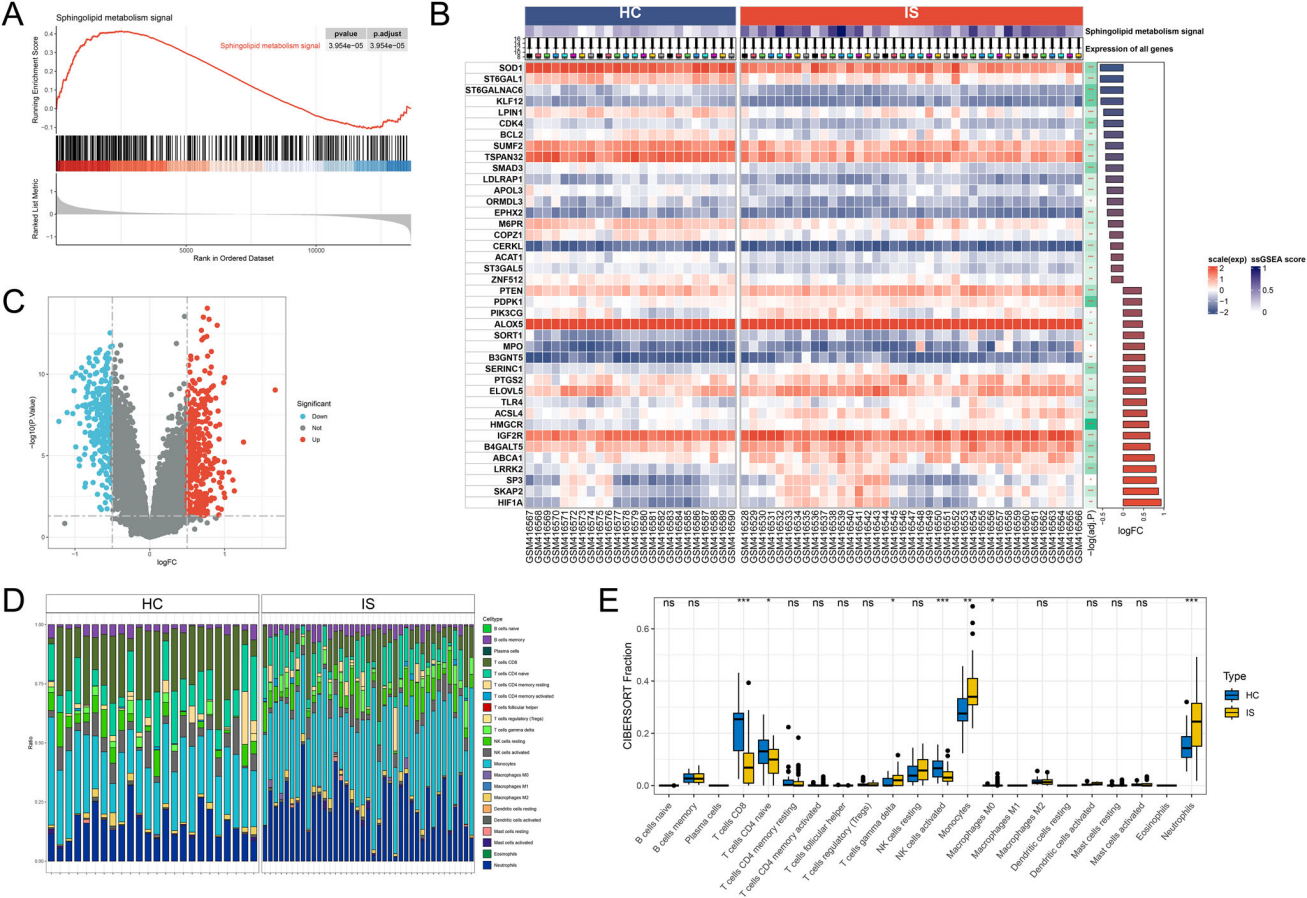
Sphingolipid metabolism significantly changes in stroke. (A) GSEA analysis, (B) top 20 genes with the most significant upregulation and downregulation fold changes, (C) differential volcano plot, (D) relative content of 22 cell types by CIBERSORT, and (E) box plots showing inter‐group differences.

**FIGURE 2:**
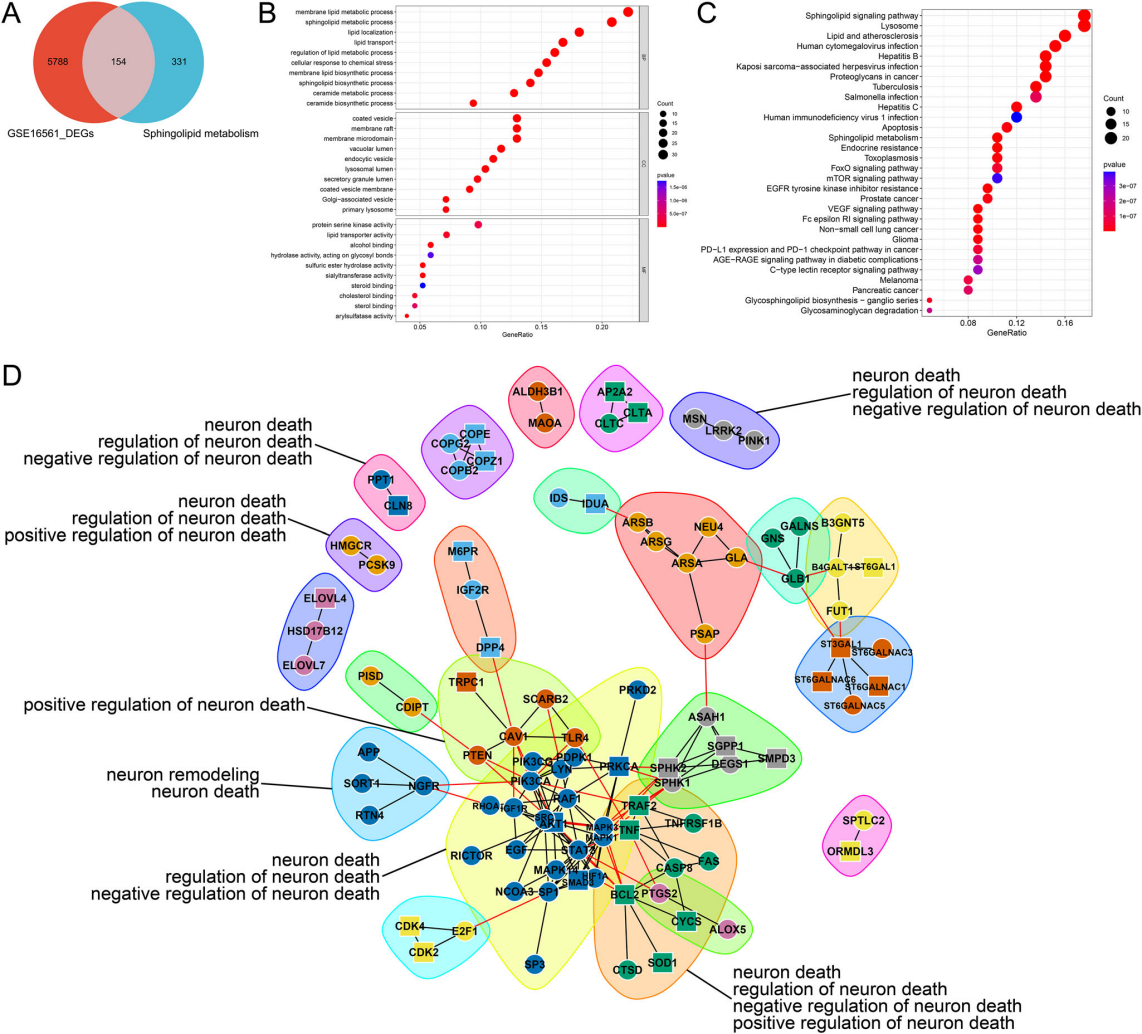
Identification genes of Sphingolipid metabolism. (A) SPM‐related genes, (B) GO enrichments, (C) KEGG enrichments, and (D) Protein interaction network.

Through retrieval and screening, we ultimately obtained 485 SPM‐related genes. Subsequent to processing the IS chip data, GSEA analysis was performed, as shown in Figure 1A. Compared to the peripheral blood of healthy individuals, the sphingolipid metabolism pathway in stroke patients exhibited significant changes. We detailed the top 20 genes with the most significant upregulation and downregulation in terms of fold change, as shown in Figure 1B. A differential analysis was then conducted, identifying 5,942 differentially expressed genes, of which 154 genes aligned with the sphingolipid metabolism pathway were categorized as SPM‐related genes, as shown in Figure 2A.

### 3.2 Immune Infiltration Analysis

We conducted CIBERSORT immune infiltration analysis, showing the relative content of 22 cell types in each sample as seen in Figure 1D, and group differences are shown in the box plot in Figure 1E. The analysis reveals that CD8 T cells, CD4 naïve T cells, and activated NK cells significantly decreased in the IS group, whereas gamma delta T cells, monocytes, and neutrophils significantly increased.

### 3.3 Enrichment Analyses of SPM‐Related Genes

To further understand the biological processes and signaling pathways involving SPM‐related genes, GO and KEGG enrichment analyses were conducted. The most significant pathway in KEGG enrichment, shown in Figure 2C, was the sphingolipid signaling pathway, with a particularly notable presence of sphingolipid metabolic processes in the BP enrichment results. This confirms the rationale behind the SPM‐related genes identified in our study. In the BP enrichment results, the ceramide metabolic process and ceramide biosynthetic process are noteworthy. Ceramide is an essential component of cell membranes and plays a crucial role in various cellular functions, including differentiation, proliferation, apoptosis, and signal transduction. In the sphingolipid pathway, sphingolipids are cleaved by enzymes to generate ceramide and phosphocholine or phosphoethanolamine. This pathway mainly occurs on the cell membrane and is crucial for the rapid increase of ceramide levels in response to external signals.

### 3.4 Protein‐Protein Interaction Network Construction and Module Analysis

In the nervous system, sphingolipids serve essential functions vital for neuron survival and signal transmission. Ceramides, a type of sphingolipid, play a vital role in regulating neuron death. To effectively understand the genetic links between sphingolipid metabolism and neuron death, we input SPM‐related genes into the STRING database to obtain their protein interaction network and performed modular analysis and biological process enrichment. Through the screening of enrichment results, we identified 19 key genes linked between sphingolipid metabolism and neuron death, which are highlighted for further analysis, as shown in Figure 2D.

### 3.5 scRNA Analysis

**FIGURE 3:**
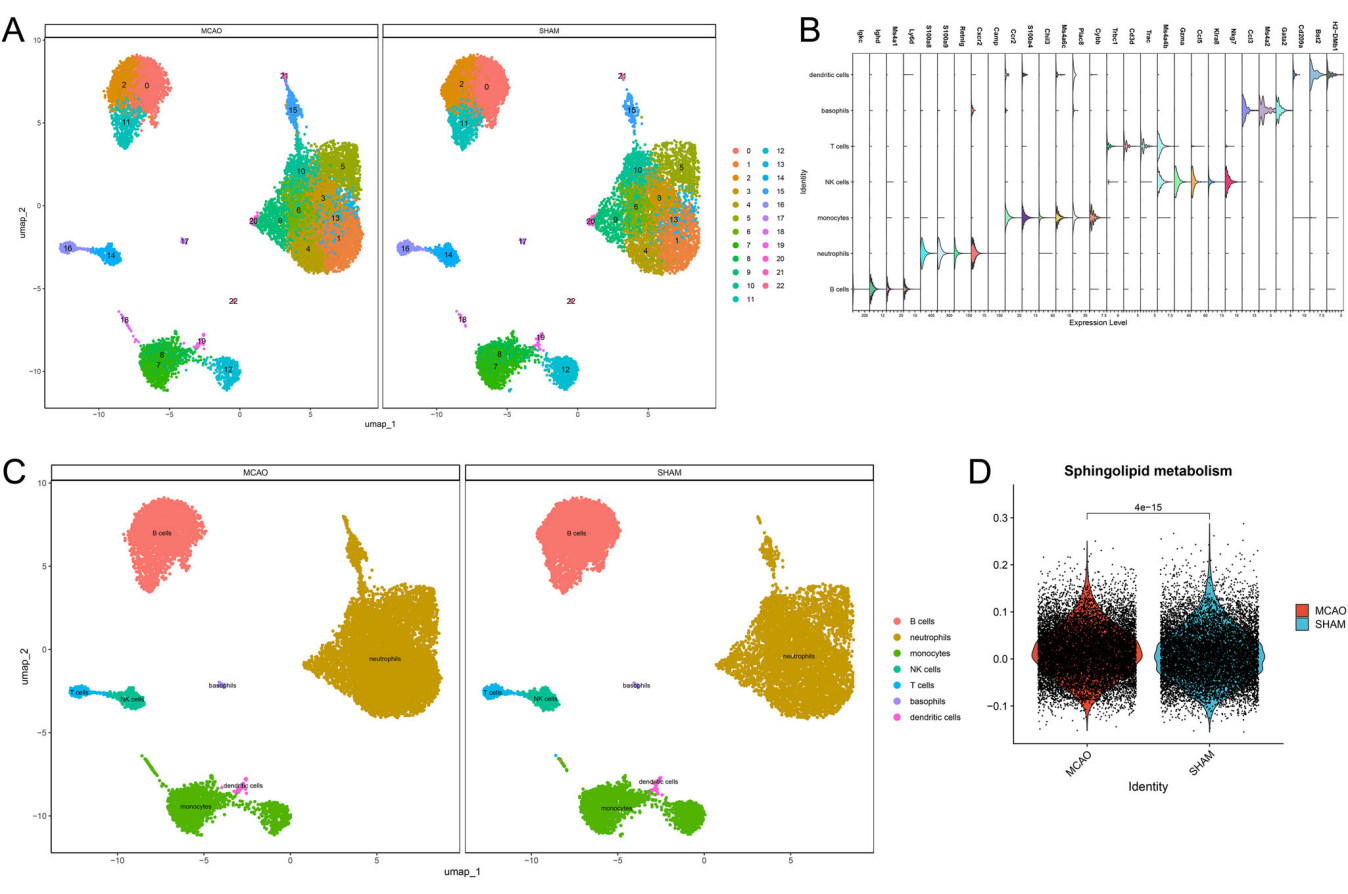
scRNA annotation and differential analysis of SPM. (A) Clustering of different cell types in the scRNA dataset by umap, (B) bubble plot of cell marker expression levels, (C) scRNA annotation, and (D) SPM scores in MCAO and Sham groups.

We acquired the MCAO mouse peripheral blood single‐cell sequencing dataset GSE225948 and performed quality control, with results shown in Figure S1. The number of PCA is determined to be 20. After a series of dimensionality reductions, UMAP visualization is shown in Figure 3A. Subsequently, cluster annotations were performed manually, with cell marker violin plots shown in Figure 3B. The final annotated UMAP plot is shown in Figure 3C. As indicated, we annotated a total of seven cell types, with the majority being neutrophils. We then scored the sphingolipid metabolism pathway in both MCAO and SHAM groups and plotted differential violin plots as shown in Figure 3D. As indicated, the sphingolipid metabolism pathway underwent significant changes after stroke onset.

### 3.6 Verifying Expression Differences of Key Genes in scRNA Data

**FIGURE 4:**
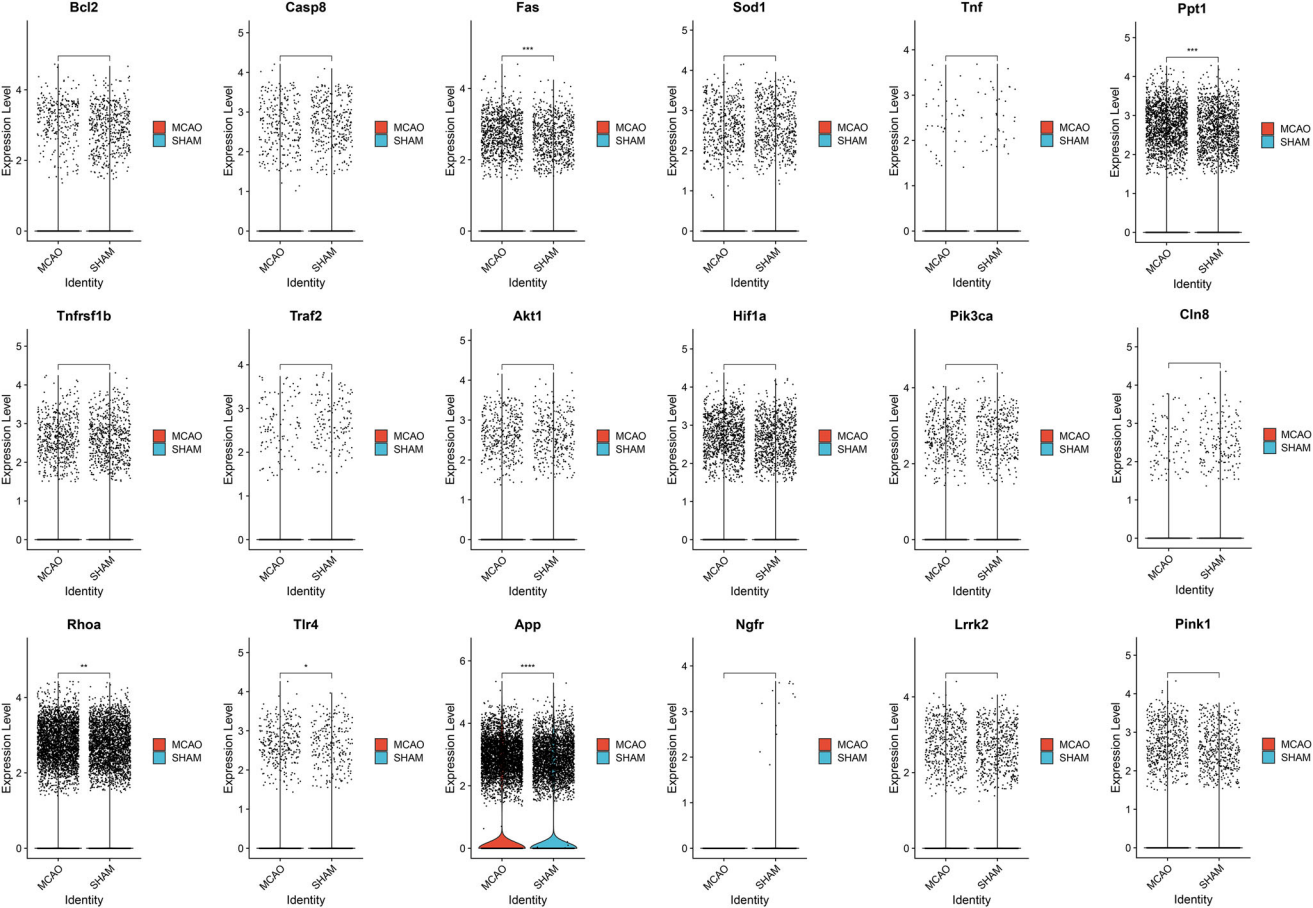
The hub genes of SPM‐related genes.

Upon filtering, we identified 19 key genes linked between sphingolipid metabolism and neuron death. Verification within the single‐cell dataset revealed differences in only 5 genes between the MCAO and SHAM groups, as shown in Figure 4. These genes are App, Fas, Rhoa, Tlr4, and Ppt1, with the App gene showing especially notable differences. These 5 genes underwent cross‐validation in both bulk and single‐cell datasets, indicating their important role in the pathophysiological changes of neuron death due to sphingolipid metabolism.

### 3.7 Analyzing Changes in Sphingolipid Metabolism From a Cellular Perspective

**FIGURE 5:**
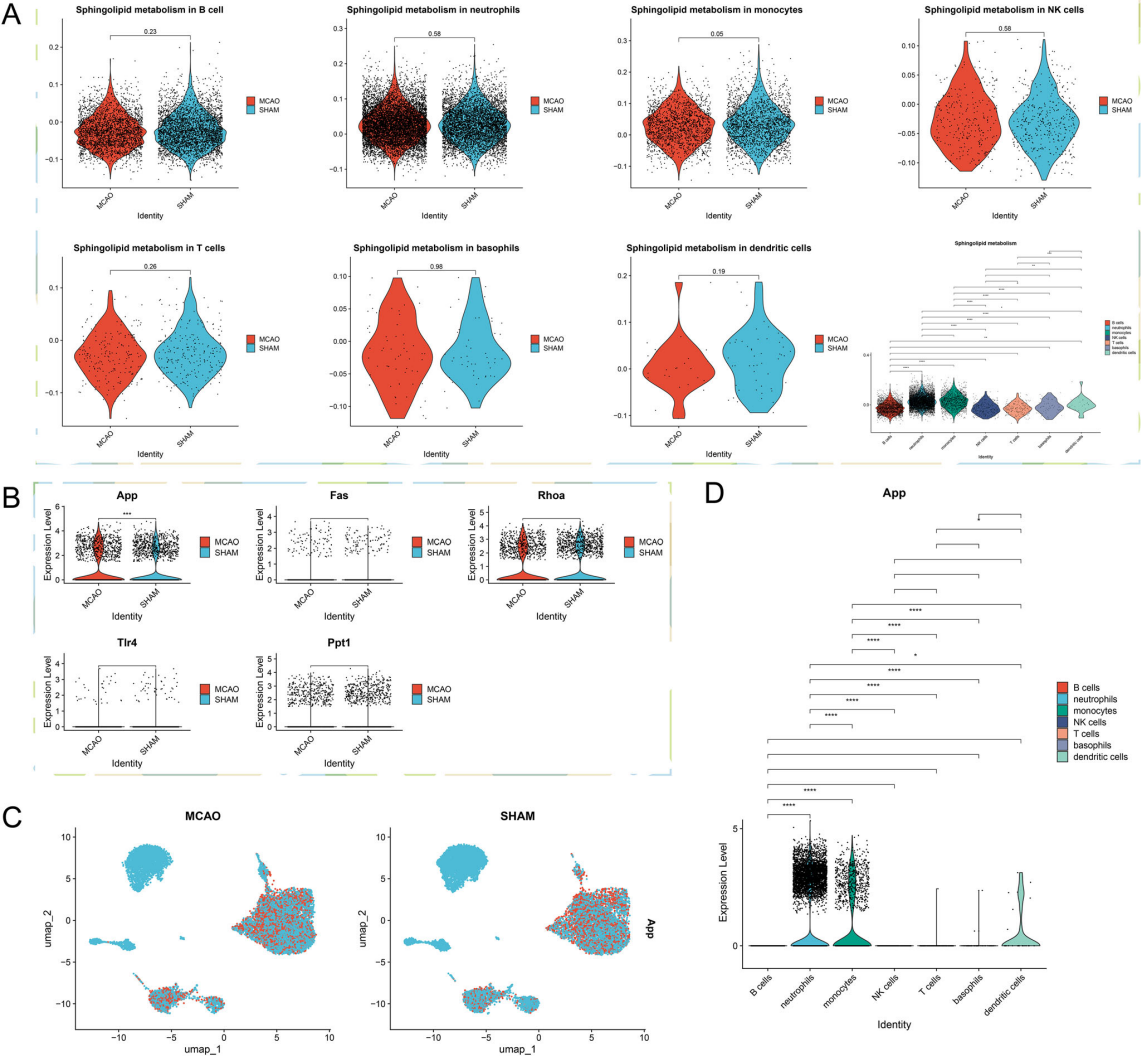
The connection between monocytes and SPM. (A) The differences of SPM in various cells, (B) differential expression of key SPM genes in monocytes, (C) the expression distribution of the App, and (D) differential expression of App in various cells.

To further observe the activation levels of sphingolipid metabolism after stroke from the cellular level, we extracted scores of sphingolipid metabolism for each cell type and conducted group comparisons between MCAO and SHAM as well as among cells, with results shown in Figure 5A. It was observed that in monocytes, the MCAO group is slightly higher than the SHAM group, with the differences barely considered statistically significant. In pairwise comparisons among cells in the MCAO group, significant sphingolipid metabolism was found in B cells, neutrophils, and monocytes. Monocytes scored the highest for sphingolipid metabolism, although neutrophils were most numerous, suggesting the sheer size of neutrophils does not indicate a higher likelihood of sphingolipid metabolism occurrence. Comparisons showed the most significant changes in sphingolipid metabolism in monocytes after stroke; hence, subsequent investigations will focus on key genes, differentiation status, and interactions between monocytes and other cells.

### 3.8 Identifying Key Genes in Monocytes for Sphingolipid Metabolism‐Induced Neuron Death Changes

From the 5 key genes identified as causing neuron death changes through sphingolipid metabolism via cross‐validation of bulk and single‐cell datasets, their differential expression was further verified in monocytes as shown in Figure 5B. Surprisingly, only App was significantly elevated in the MCAO group, showing remarkable statistical significance compared to the SHAM group. To visually illustrate App gene expression across cells, we plotted Figure 5C, with App predominantly expressed in neutrophils and monocytes. To delineate cell‐type‐specific expression differences, violin plots were drawn for pairwise comparisons as shown in Figure 5D. This illustration elucidates that in the MCAO group, App is expressed in neutrophils, monocytes, T cells, basophils, and dendritic cells, but most abundantly in neutrophils and monocytes, and significantly higher in neutrophils. This cell‐specific expression of the App gene warrants further exploration.

### 3.9 Key Gene Related Analysis

**FIGURE 6:**
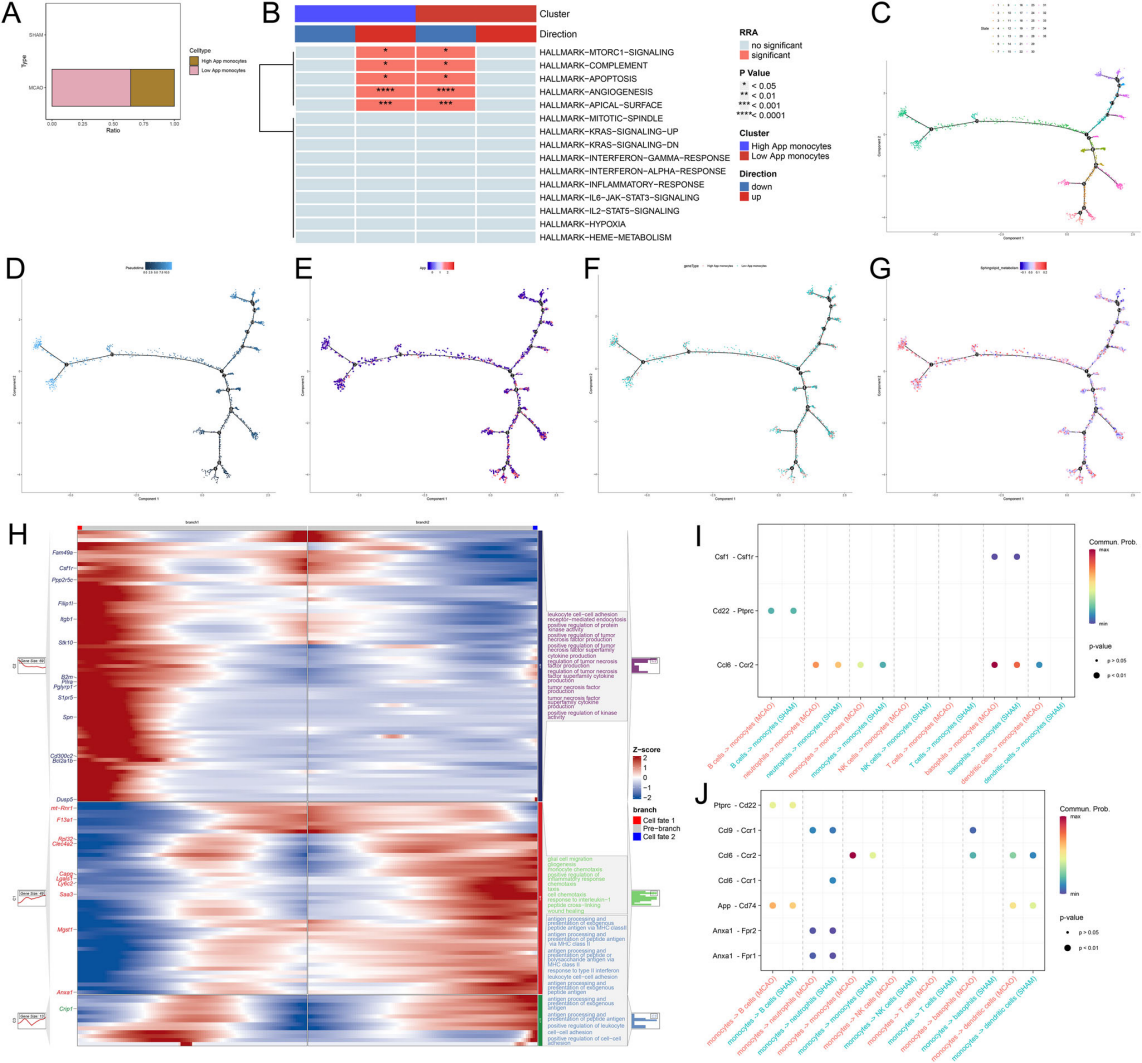
The related analysis of App. (A) High App‐monocytes group and low App‐monocytes group based on median App gene expression, (B) irGSEA analysis, (C, D) differentiation status of monocytes, (E) App expression along the pseudo‐time in monocytes, (F) high App‐monocytes group and low App‐monocytes group along the pseudo‐time in monocytes. (G) SPM Score along the pseudo‐time in monocytes, (H) the cell fate and BP enrichments of monocytes, and (I, J) cellchat analysis.

To comprehensively understand the phenotypic changes potentially impacted by the App gene in monocytes, we isolated monocytes in the MCAO group, and divided them into high App monocytes and low App monocytes based on median App gene expression, categorized as shown in Figure 6A. Subsequently, the RRA (robust rank aggregation) algorithm of the irGSEA package was used for an integrated score. The RRA algorithm evaluates differential analyses from four algorithms, “AUCell,” “UCell,” “singscore,” and “ssgsea,” aggregating significantly enriched gene sets in most algorithms. This greatly mitigates error introduced by singular algorithms, enabling a more comprehensive and reliable biological information analysis. Based on this algorithm, a heatmap was drawn for an intuitive display of progression as shown in Figure 6B. Results revealed 5 HALLMARK pathways with differences between high and low App expression groups, with pathway activation levels positively correlated with App gene expression. These pathways are MTORC1 SIGNALING for protein synthesis and autophagy regulation, COMPLEMENT crucial for immune defense and maintaining health, APOPTOSIS for cellular turnover, ANGIOGENESIS indispensable in wound healing, and APICAL SURFACE vital for proper structural and functional localization.

### 3.10 Pseudotime Analysis

We extracted monocytes and conducted pseudotime analyses to further understand their differentiation status. Constructing a developmental trajectory with pseudotime, as shown in Figure 6C,D, identified 17 branching points and 35 stages. These results suggest a diverse and dynamic differentiation process for monocytes post‐stroke. App gene expression changes along the pseudotime developmental trajectory were evaluated as shown in Figure 6E,F. These plots indicate that cells with high App expression are relatively numerous early in monocyte development but become sparse towards the end. The sphingolipid metabolism score distribution was assessed as shown in Figure 6G, showing high metabolic scoring early in the developmental tree, with significant pathway score differences post‐branch 1 between two cellular fates, one showing notably higher high sphingolipid metabolism scores. Based on results, further branch point analysis focused on branch 1, clustering genes with similar expression patterns showcasing most notable changes, as presented in Figure 6H. Cluster 2 contained the most genes with enrichment results, including leukocyte cell‐cell adhesion, cytokine production, tumor necrosis factor regulation, etc. Cluster 1 genes were enriched in monocyte chemotaxis, cell chemotaxis, positive regulation of inflammatory responses, etc. Cluster 3 genes were enriched in antigen processing and presentation.

### 3.11 CellChat Analysis

Using all cell types, the communication between cells was investigated with monocytes as both recipient and source cells in MCAO and SHAM groups, as shown in Figures 6I and 6J. Pathways such as Ccl6‐Ccr1 showed significant differences. Unexpectedly, the App‐Cd74 pathway, which connects monocytes as the source cell with B cells as the recipient, also presented differences, being less activated in the SHAM group than in the MCAO group.

### 3.12 PCR Validation In Vivo

**FIGURE 7:**
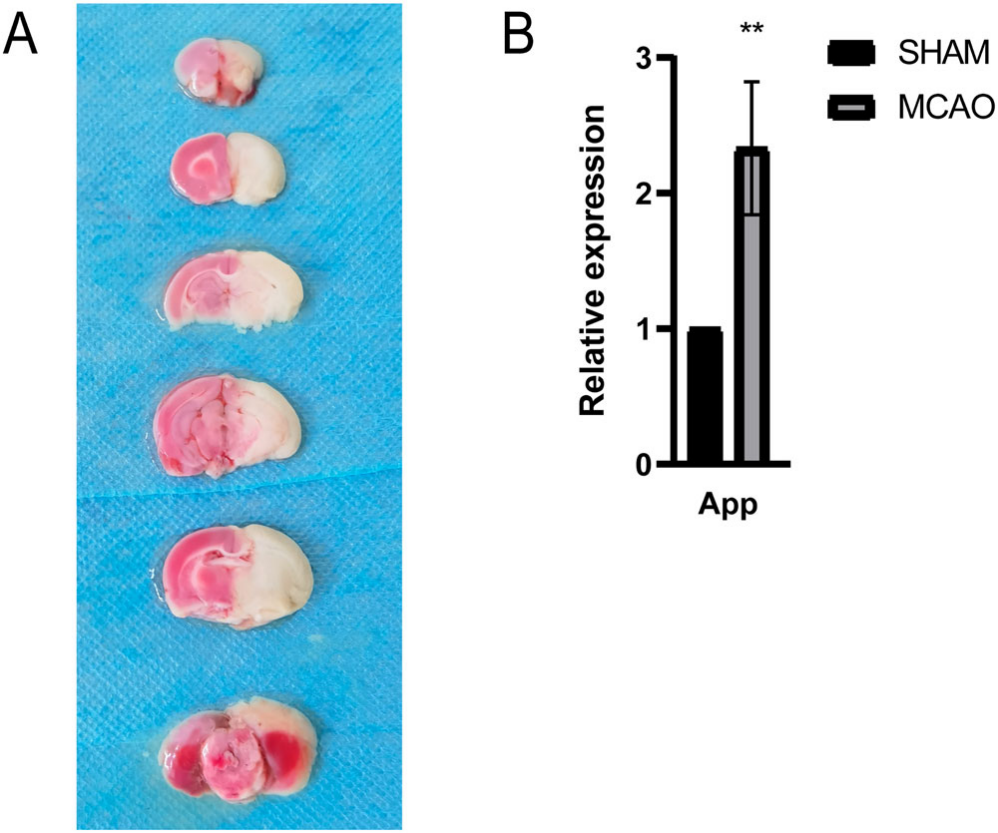
In vivo experimental verification. (A) TTC staining of MCAO and SHAM groups and (b) mRNA expression levels of hub genes in MCAO and SHAM groups.

To ensure rigorous data, we successfully prepared MCAO rats, as shown in Figure 7A. Unstained areas indicate cerebral infarction regions. Subsequent PCR validation of blood samples, shown in Figure 7B, indicated significantly higher App gene expression in the MCAO group compared to the SHAM group.

## 4 Discussion

This study analyzed human peripheral blood chip data to determine that sphingolipid metabolism undergoes significant changes after ischemic stroke (IS). Subsequently, using a series of algorithms, we identified bridge genes linking sphingolipid metabolism and neuron death. To delve further from a cellular level, we employed single‐cell sequencing data from the peripheral blood of MCAO mice and discovered that changes in sphingolipid metabolism predominantly occur in monocytes. We then identified App as a key bridging gene between sphingolipid metabolism and neuron death and explored various mechanisms of the App gene.

Inference from cell algorithms on peripheral blood chips showed a significant increase in monocytes after the occurrence of IS. Numerous studies indicate that monocytes play a crucial role in IS onset and progression. IS further disrupts the blood‐brain barrier, allowing substantial monocyte infiltration into the brain parenchyma, where they differentiate into pro‐inflammatory or anti‐inflammatory phenotypes (Goh et al. 2024). The pro‐inflammatory phenotype secretes cytokines that induce cerebral edema, exacerbating brain damage, while the anti‐inflammatory phenotype secretes growth factors that promote neurogenesis and angiogenesis, contributing to the repair of the blood‐brain barrier post‐injury (Blank‐Stein and Mass 2023). Research on therapeutic targets concerning monocyte reprogramming, such as soluble PD‐L1, demonstrates potential in preventing cerebral edema and promoting recovery post‐IS (Kim et al. 2024).

The higher sphingolipid metabolism score in monocytes of IS patients compared to SHAM suggests a pivotal role for these cells in stroke‐induced inflammation. Monocytes are central to the immune response in stroke, and their altered sphingolipid metabolism could exacerbate inflammation and tissue damage. This finding underscores the need to explore monocyte‐specific metabolic pathways as potential therapeutic targets. Understanding how these metabolic changes in monocytes influence their function during stroke could provide novel insights into inflammation control in stroke patients.

Sphingolipids, as essential components of cell membranes, play a significant role in various cellular processes, including cell signaling, apoptosis, and inflammation, which are central to the pathophysiology of stroke. These bioactive lipids, particularly ceramide and sphingosine‐1‐phosphate (S1P), have been identified as key mediators in the regulation of these processes. Ceramide is known for its role in promoting apoptosis, a form of programmed cell death, which can be detrimental in the context of stroke as it may lead to the loss of neuronal cells. On the other hand, S1P is involved in cell survival signaling and has been implicated in inflammatory responses, which are crucial in the acute phase of stroke (Maceyka and Spiegel 2014; Tringali and Giussani 2022). Recent advancements in sphingolipidomics and the development of animal models have provided deeper insights into the molecular mechanisms by which sphingolipids influence inflammation and immune responses. These studies have highlighted the potential of targeting sphingolipid metabolism as a therapeutic strategy for inflammatory disorders, including those related to stroke. The compartmentalization and translocation of sphingosine kinases, which are responsible for the production of S1P, have been shown to affect both the signaling output and the metabolic regulation of sphingolipids, further emphasizing their role in cellular processes relevant to stroke (Siow and Wattenberg 2011; Velazquez et al. 2021). Moreover, the involvement of sphingolipids in neuroinflammation and neurodegeneration has been increasingly recognized, particularly in the context of the central nervous system (CNS). Sphingolipids and their metabolic intermediates orchestrate various physiological processes in the CNS and participate in neuroinflammatory and neurodegenerative disorders. This understanding opens up new avenues for therapeutic interventions targeting sphingolipid pathways to mitigate the effects of stroke and improve outcomes for patients suffering from such cerebrovascular events (Ayub et al. 2021; Gualtierotti et al. 2017).

Ceramide, a central molecule in sphingolipid metabolism, plays a crucial role in regulating apoptosis pathways, particularly in neuronal cells. The involvement of ceramide in apoptosis is well‐documented, with studies highlighting its function as a second messenger in apoptotic signaling pathways. For instance, ceramide has been shown to induce apoptosis in neuronal cells by influencing mitochondrial pathways and activating caspases, which are critical components of the apoptotic machinery (Zhang et al. 2012). Moreover, the regulation of ceramide levels is crucial for maintaining cellular homeostasis, and disruptions in ceramide metabolism can lead to neurodegenerative disorders. In particular, ceramide has been implicated in the pathogenesis of Alzheimer's disease, where it contributes to neurodegeneration and amyloidogenesis. The interplay between ceramide and amyloid beta protein is thought to exacerbate mitochondrial dysfunction, further promoting neuronal apoptosis (Chakrabarti et al. 2016). In addition to its role in neurodegenerative diseases, ceramide is also involved in the cellular response to oxidative stress. For example, in neuronal cells, oxidative stress can lead to increased ceramide levels, which in turn promote apoptosis through the activation of specific signaling pathways, such as the p38 MAPK pathway. This highlights the importance of ceramide in mediating the cellular response to stress and its potential as a therapeutic target for protecting neurons from apoptosis (Tu et al. 2016).

In monocytes, we identified the key gene App, which links sphingolipid metabolism to neuron death. The connection between sphingolipid metabolism and neuronal cell death is a significant area of research, particularly in the context of neurodegenerative diseases such as Alzheimer's disease (AD). The gene App, known for its role in amyloid precursor protein processing, is intricately linked to these processes. Sphingolipids, including ceramide and sphingosine‐1‐phosphate (S1P), are crucial in cell signaling pathways that regulate cell death and survival. Ceramide, for instance, has been implicated in promoting neurodegeneration and amyloidogenesis in AD. It is known to interact with amyloid beta (Aβ) peptides, contributing to mitochondrial dysfunction and neuronal apoptosis (Chakrabarti et al. 2016). Moreover, the deregulation of sphingolipid metabolism, particularly the balance between ceramide and S1P, is a critical factor in AD pathogenesis. Sphingosine kinase‐1 (SPK1) and sphingosine kinase‐2 (SK2) are enzymes that regulate this balance. SPK1, for example, has been shown to protect neurons from Aβ‐induced toxicity by modulating mitochondrial pathways, thus highlighting its potential as a therapeutic target for AD (Yang et al. 2014). Similarly, SK2 has been reported to influence amyloid deposition and neuroprotection, suggesting that alterations in sphingolipid metabolism can significantly impact AD progression (Lei et al. 2019). Furthermore, the role of sphingolipids in neurodegeneration is not limited to AD. In Parkinson's disease‐related depression (PDD), altered lipid and glucose metabolism have been identified as contributing factors to cellular injury and oxidative stress. This highlights the broader implications of sphingolipid metabolism in various neurodegenerative conditions (Dong et al. 2018). Understanding the molecular mechanisms linking sphingolipid metabolism with neuronal cell death could provide new insights into therapeutic strategies for these debilitating diseases. The differential expression of key genes such as App highlights their potential roles in stroke pathogenesis. While its role in stroke is less understood, its upregulation suggests a possible neurotoxic function in the ischemic environment. Exploring the functions of the gene in the context of stroke could uncover new pathways involved in disease progression and identify novel therapeutic targets.

Meanwhile, our research findings suggest that our irGSEA analysis suggested a potential association between high App expression and angiogenesis pathways, which warrants further investigation. The regulation of angiogenesis is a complex process involving numerous genes and signaling pathways. Our findings suggest that the gene App may play a role in this intricate network. Angiogenesis, the formation of new blood vessels, is crucial for various physiological processes, including wound healing and embryonic development, as well as pathological conditions such as cancer and diabetic retinopathy. The role of App in angiogenesis can be linked to its involvement in Alzheimer's disease, where vascular perturbations are a critical component of the disease's pathogenesis. In Alzheimer's, amyloid beta (Aβ) accumulation is associated with increased angiogenesis and vascular permeability, suggesting that App, which is involved in Aβ production, may influence angiogenic processes (Jefferies et al. 2013). This connection highlights the potential for App to affect angiogenesis through its impact on amyloidogenic pathways. Furthermore, the role of App in angiogenesis is supported by studies on other angiogenesis‐related genes. For instance, the expression of angiogenesis markers such as VEGF and HSP70 has been observed in various cancers, indicating that genes involved in angiogenesis are often upregulated in tumor environments (Schoedel et al. 2021). This suggests that App, through its regulatory functions, might interact with these pathways to modulate angiogenic responses. Additionally, the involvement of App in angiogenesis is further corroborated by research on diabetic retinopathy, where angiogenesis plays a significant role in disease progression. In this context, genes like miR‐210 have been shown to regulate endothelial cell proliferation under high‐glucose conditions, a process that App might influence through its regulatory network (Yin et al. 2020). This highlights the potential for App to serve as a therapeutic target in diseases characterized by dysregulated angiogenesis. In summary, the gene App appears to be a significant player in the regulation of angiogenesis, with implications for both physiological and pathological processes. Its interactions with other angiogenesis‐related genes and pathways underscore its potential as a target for therapeutic interventions in diseases where angiogenesis is a key factor.

The pseudotime analysis revealing higher App expression in early monocyte differentiation stages suggests a role for App in the initial inflammatory response post‐stroke. This could indicate that App influences monocyte function during the acute phase of stroke, potentially affecting inflammation dynamics. Further studies are needed to elucidate the exact function of App in monocyte differentiation and its impact on stroke outcomes.

The CellChat analysis showing differences in communication pathways, including App‐Cd74 between monocytes and B cells, indicates altered immune interactions in stroke. The App‐Cd74 axis provides critical insights into the altered immune interactions that occur in various pathological conditions, including stroke. The App‐Cd74 pathway is particularly significant as it highlights the complex interplay between monocytes and B cells, which are crucial components of the immune system. This pathway's involvement in stroke suggests that immune cell communication is disrupted, potentially contributing to the disease's progression and severity. In the context of stroke, the blood‐brain barrier (BBB) plays a pivotal role in maintaining the brain's microenvironment. However, ischemic stroke can lead to profound immune responses that affect the BBB's integrity, as peripheral immune cells interact with the BBB, influencing both its disruption and repair processes (Li et al. 2018). The interaction between immune cells and the BBB is a double‐edged sword, as it can lead to both detrimental and beneficial outcomes depending on the context and timing of the immune response. Furthermore, the role of circulating monocytes in the neuroinflammatory processes associated with stroke is well‐documented. These cells can infiltrate the brain and interact with resident glial cells, such as microglia and astrocytes, contributing to the inflammatory milieu that characterizes stroke pathology (Greenhalgh et al. 2020). The communication between monocytes and glial cells is crucial for understanding how immune responses are modulated during stroke and how they can be targeted for therapeutic interventions. Additionally, the interaction between monocytes and other immune cells, such as B cells, through pathways like App‐Cd74, underscores the complexity of the immune network in stroke. This pathway may influence the immune cell dynamics within the stroke microenvironment, potentially affecting the overall immune response and the progression of brain injury (Chen et al. 2024). Understanding these interactions at a molecular level could pave the way for novel therapeutic strategies aimed at modulating immune responses to improve stroke outcomes. In conclusion, the App‐Cd74 communication pathway between monocytes and B cells is a crucial component of the altered immune interactions observed in stroke. By elucidating the roles of various immune cells and their communication pathways, researchers can better understand the pathophysiology of stroke and develop targeted therapies to mitigate its impact.

Despite the comprehensive nature of our analysis, this study has several limitations that should be acknowledged. Firstly, the associative relationships identified through bioinformatics analyses, while insightful, do not establish causality. The predicted roles of key genes like App and the highlighted cellular pathways require further functional validation through in vitro and in vivo experimental interventions, such as gene knockout or overexpression models. Secondly, our study integrates data from multiple species, including human bulk transcriptomic data, mouse single‐cell sequencing data, and in vivo validation in rats. Although this multi‐species approach strengthens the translational potential of our findings, it also introduces potential limitations due to species‐specific differences in immune response, gene expression, and sphingolipid metabolism. The translational relevance of mechanisms identified in rodent models to human ischemic stroke pathophysiology warrants careful interpretation and further investigation. Thirdly, the primary focus of our analysis was on peripheral blood samples. While peripheral immune cells play a crucial role in stroke pathogenesis and are readily accessible for clinical translation, they may not fully capture the complex molecular interactions occurring within the brain parenchyma, such as those between neurons, microglia, and astrocytes. Future studies incorporating brain tissue samples will be essential to provide a more complete picture of the role of sphingolipid metabolism in central neuron death. Finally, the sample size of the single‐cell RNA sequencing dataset, while valuable for exploring cellular heterogeneity, is relatively modest. A larger cohort would enhance the statistical power and robustness of the findings, particularly for identifying rare cell subtypes and subtle transcriptional changes. Addressing these limitations in future work will be important for solidifying the therapeutic potential of targeting sphingolipid metabolism and its key regulators like App in ischemic stroke.

In conclusion, this study provides valuable insights into the role of sphingolipid metabolism in ischemic stroke, highlighting potential therapeutic targets and pathways for further exploration. While the findings are promising, the limitations, such as the sample size in single‐cell data, underscore the need for additional research. Future studies should focus on functional validation of these genes and pathways, as well as exploring the immunometabolic interactions in stroke. This comprehensive approach could lead to novel therapeutic strategies to improve stroke outcomes.

## Author Contributions

ZC and FX contributed to the searched the literature and designed the study. SH and QC contributed to perform the statistical analysis. JZ and LS contributed to the acquisition of data. SH assisted in the data processing and performed the functional analysis. ZC and FX reviewed the data and drafted the manuscript. HA, JG, and JX assisted in editing the manuscript. All authors have read and approved the final manuscript.

## Funding

This study was supported by Henan Center for Outstanding Overseas Scientists, Number: GZS2022019; Henan Province International Science and Technology Cooperation Project, Number: 242102520044; Henan Province Medical Science and Technology Research Program Joint Construction Project, Number: LHGJ20210061.

## Ethics Statement

The authors have nothing to report.

## Supporting information




**Supplementary Figure**: brb371172‐sup‐0001‐FigureS1.png

**Supplementary Table**: brb371172‐sup‐0002‐TableS1.docx

## Data Availability

The datasets GSE16561 (https://www.ncbi.nlm.nih.gov/geo/query/acc.cgi?acc = GSE16561) and GSE225948 (https://www.ncbi.nlm.nih.gov/geo/query/acc.cgi?acc = GSE225948), used and/or analyzed during the current study are available from the corresponding author on reasonable request.
