# Supplementary figures and images for: The Role of Sphingolipid Metabolism and Neuron Death in Ischemic Stroke: A New Perspective from Bioinformatics
Source: Brain Behav. 2025 Dec 31;16(1):e71172. doi: 10.1002/brb3.71172 (PMC12755557; doi:10.1002/brb3.71172)

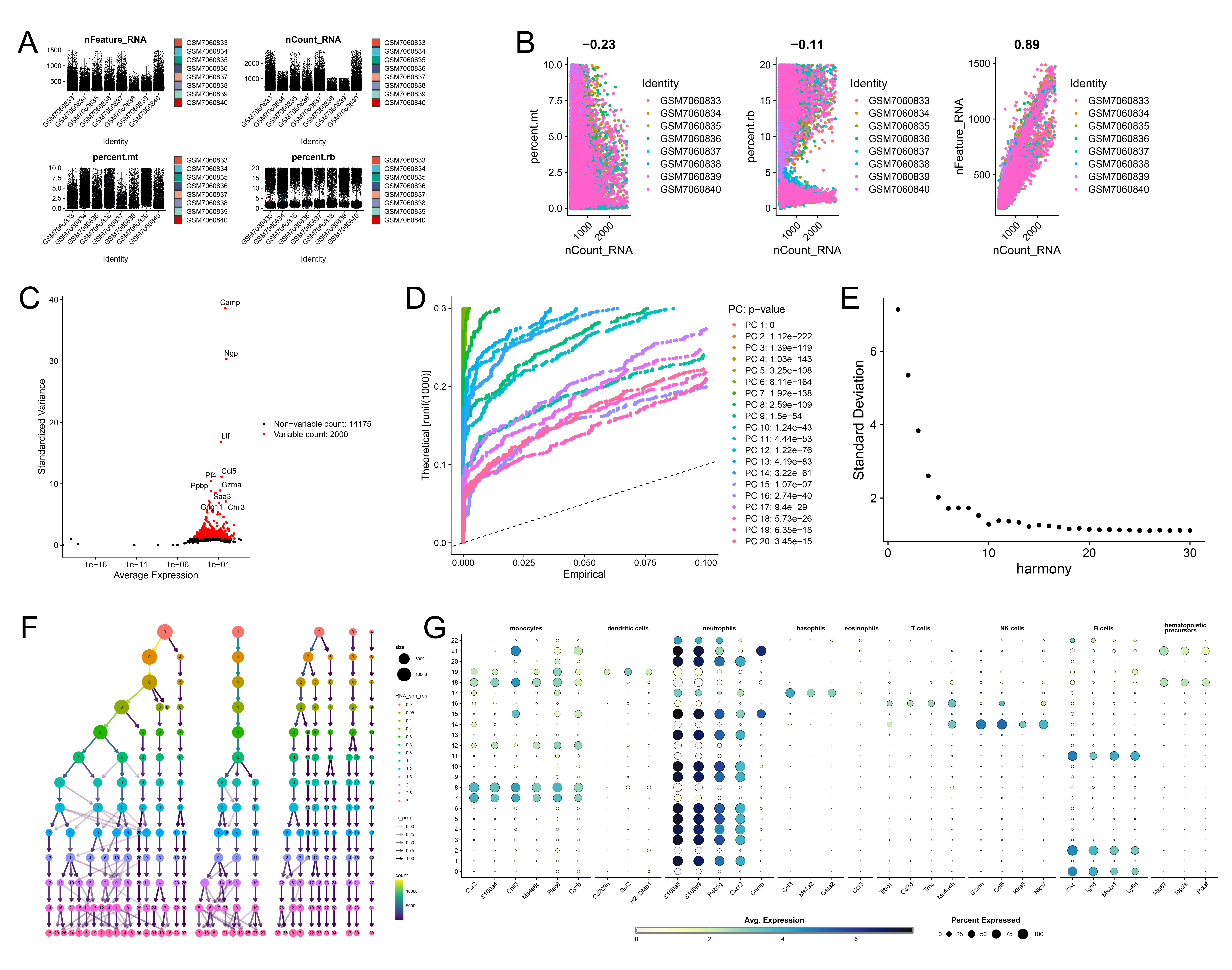

Supplement: Supplementary file 1 — Supplementary Figure: brb371172‐sup‐0001‐FigureS1.png [file BRB3-16-e71172-s002.png]
