# Supplementary material for: The Role of Sphingolipid Metabolism and Neuron Death in Ischemic Stroke: A New Perspective from Bioinformatics
Source: Brain Behav. 2025 Dec 31;16(1):e71172. doi: 10.1002/brb3.71172 (PMC12755557; doi:10.1002/brb3.71172)
Supplement: Supplementary file 2 — Supplementary Table: brb371172‐sup‐0002‐TableS1.docx [file BRB3-16-e71172-s001.docx]

Supplement Table 1 Primers for detection of target genes by Real-time PCR

| Primer | Sequence | Amplicon Size |
| --- | --- | --- |
| App-F | GGAGCAGAACTACTCCGACG | 221 |
| App-R | CGTCGACAGGCTCAACTTCA |  |
| GAPDH-F | TGTGTCCGTCGTGGATCTGA | 150 |
| GAPDH-R | TTGCTGTTGAAGTCGCAGGAG |  |
